# Medicines, Diseases, Indications, and Contraindications (MeDIC): a foundational resource to support drug repurposing

**DOI:** 10.1093/nar/gkaf1312

**Published:** 2025-12-12

**Authors:** Marcello DeLuca, Nico Matentzoglu, Elliott Sharp, Jane Li, Charlie Hempstead, May Lim, Piotr Kaniewski, E Kathleen Carter, Kushal Koirala, Elvin Ding, Laurens Vijnck, Pascal Brokmeier, Sabrina Toro, Kevin Schaper, Jacques Vergine, Olivia Li, Tudor I Oprea, David C Fajgenbaum, Christopher Bizon, Melissa Haendel, Alexander Tropsha

**Affiliations:** Division of Chemical Biology and Medicinal Chemistry, University of North Carolina Eshelman School of Pharmacy, Chapel Hill, NC 27599, United States; Semanticly, Athens 10563, Greece; Every Cure, Philadelphia, PA 19104, United States; Every Cure, Philadelphia, PA 19104, United States; Every Cure, Philadelphia, PA 19104, United States; Every Cure, Philadelphia, PA 19104, United States; Every Cure, Philadelphia, PA 19104, United States; Renaissance Computing Institute, Chapel Hill, NC 27517, United States; Division of Chemical Biology and Medicinal Chemistry, University of North Carolina Eshelman School of Pharmacy, Chapel Hill, NC 27599, United States; Division of Chemical Biology and Medicinal Chemistry, University of North Carolina Eshelman School of Pharmacy, Chapel Hill, NC 27599, United States; Every Cure, Philadelphia, PA 19104, United States; Every Cure, Philadelphia, PA 19104, United States; Department of Genetics, University of North Carolina School of Medicine, Chapel Hill, NC 27599, United States; Department of Genetics, University of North Carolina School of Medicine, Chapel Hill, NC 27599, United States; Every Cure, Philadelphia, PA 19104, United States; Division of Chemical Biology and Medicinal Chemistry, University of North Carolina Eshelman School of Pharmacy, Chapel Hill, NC 27599, United States; Expert Systems, Inc., Dover, DE 19901, United States; Every Cure, Philadelphia, PA 19104, United States; Perelman School of Medicine at the University of Pennsylvania, Philadelphia, PA 19104, United States; Renaissance Computing Institute, Chapel Hill, NC 27517, United States; Department of Genetics, University of North Carolina School of Medicine, Chapel Hill, NC 27599, United States; Division of Chemical Biology and Medicinal Chemistry, University of North Carolina Eshelman School of Pharmacy, Chapel Hill, NC 27599, United States; Renaissance Computing Institute, Chapel Hill, NC 27517, United States

## Abstract

Drug databases typically aim to provide reference information on medications and their uses but often lack strict definitions of the terms *drug* (e.g. approved or a clinical candidate) or *disease*, and do not focus on any specific context of use. The recent emergence of biomedical knowledge graphs, which integrate diverse biomedical data into a contiguous, harmonized knowledge network, has enabled innovation in drug repurposing (identification of novel uses of existing drugs). This objective has created a new set of requirements and challenges for drug databases to be used for generating high-confidence, testable drug repurposing hypotheses. To address this challenge, we have developed MeDIC as an open, foundational database built from government regulatory sources only, which comprises highly curated lists of drugs (including combination therapies), diseases, indications (i.e. drug approvals to treat specific diseases), contraindications, and additional metadata. MeDIC allows for easy maintainability, open-source adaptability, and ongoing updates concordant with updates of primary sources. To facilitate downstream use, MeDIC is provided in a tabulated format, and each drug, disease, indication, or contraindication entry is mapped to multiple ontologies. We offer MeDIC as a web-based, freely accessible (https://medic.renci.org), downloadable (including lists and source code), searchable, and machine learning-friendly resource for patients, providers, and researchers.

## Graphical Abstract

**Graphical Abstract:**
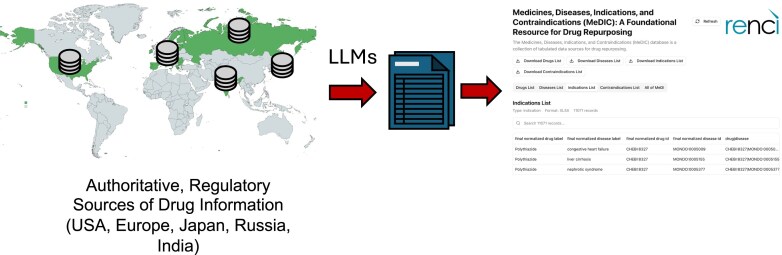

## Introduction

Driven by advances in biomedical databases and AI approaches, there is an increasing effort to apply AI to the challenge of drug repurposing, implementing the new concept of computational pharmacophenomics [1]. Drug repurposing is the practice of intentional, data-driven research into novel applications of an already-approved medication [2]. Compared to traditional drug discovery processes, which can take up to 10 years [3] and up to $2.5bn [4], drug repurposing can significantly reduce the time and cost required for a drug to reach patients [5]. The benefits of repurposing are reflected in the prevalence of off-label prescriptions in the medical community, with up to 20%–32% of prescriptions in the USA written for indications not approved by the FDA [6]. Repurposing is particularly valuable in the rare disease space, where small patient populations make it economically challenging to pursue approvals. While individually rare, such diseases contribute significantly to overall global morbidity and mortality, in aggregate impacting >300M people worldwide [7]. Overall, successful off-label uses can significantly elevate the standard of care for patients with no other treatment options.

To support intelligent generation of novel testable drug repurposing hypotheses, our team has been developing approaches that rely on a data structure called a knowledge graph (KG) [8], which integrates, harmonizes, and links biomedical concepts and the relationships between them from diverse knowledge sources. The concepts and relationships in KGs come from a variety of sources, including high-quality curated databases [9], ontologies [10], and biomedical literature [11] using technologies like named entity recognition [12] and special data ingestion and harmonization protocols like ORION (https://github.com/RobokopU24/ORION). The use of KGs for mechanistic investigations of drug activity and drug repurposing has been described in detail elsewhere [13–15]. Briefly, key approaches include querying for the relationships connecting drug to disease and forming treatment hypotheses [16], graph embedding [17–19], and machine learning and artificial intelligence (ML/AI) methods [20–24].

Recent advances in the application of ML/AI to biomedical KGs for the generation of drug repurposing predictions have highlighted the need for high-quality data [25], especially concerning which drugs are approved for use and by which authorities; which diseases are good candidates for repurposing; which drugs are currently known to treat which diseases; and which drug–disease pairs represent a contraindication. Multiple existing databases (e.g. DrugBank [26], Drug Central [27], Physician’s Desk Reference [28]) contain elements of this information. However, our team led by Every Cure (https://everycure.org/), a non-profit dedicated to creating a robust and open scientific platform to advance drug repurposing, found those sources to be either incomplete (e.g., missing indications or focusing only on a single geographic region of the world) or inadequate for pragmatic reasons such as lack of open access. Extracting requisite data from multiple sources and combining it into a single source of ground truth is a complex challenge due to the range of formats and languages, difficulties in entity linking and cross-list harmonization, and a need for validation against original federal sources. Furthermore, data extraction from existing databases requires substantial upkeep and extensive manual review every time a source is updated. Regular updates are essential in the current pharmaceutical landscape, where new drugs are discovered and registered continuously, new diseases are identified as distinct and treatable, and approvals for drug indications are constantly evolving.

**Figure 1.**
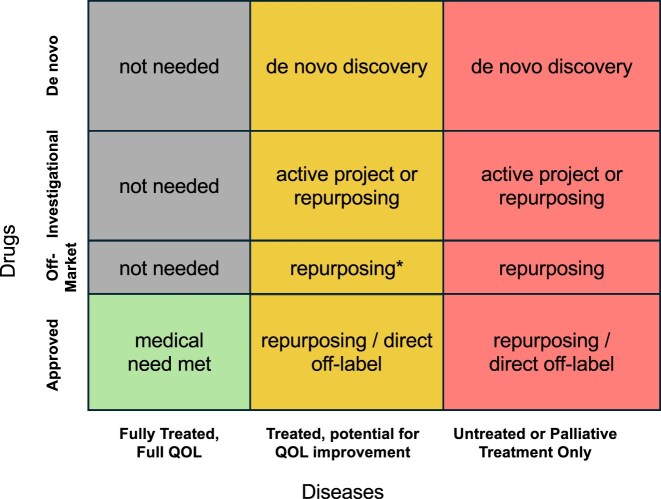
Types of known and unknown drug–disease relationships highlighting currently addressed [effective pharmacotherapeutic options exist that result in full quality of life (“QOL”), green areas]; not prioritized for drug development or repurposing (gray areas); partially addressed where new discovery or development, including repurposing, efforts are justified (yellow areas); and especially, completely unmet medical need where the only treatment options are palliative (often rare diseases) and where drug repurposing is most needed to expeditiously address patient needs (red areas). Drugs are additionally stratified according to four categories. “Approved” drugs are fully approved and have available marketed formulations. “Off-market” drugs are approved but may have been pulled from the market due to safety concerns for their original application; they have better understood safety profiles than new drugs, but marketed formulations are not available. “Investigational” drugs are under active development and may have already surpassed phase 1 clinical trials, substantially reducing the barrier to use. Lastly, “*de novo*” drugs are new drug development projects with no pre-existing evidence of safety or efficacy. *Repurposing for already treated diseases with drugs that are off-market for safety reasons is likely more difficult to justify than for conditions with no treatment options.

The MATRIX project, currently under development at Every Cure (https://everycure.org/every-cure-to-receive-48-3m-from-arpa-h-to-develop-ai-driven-platform-to-revolutionize-future-of-drug-development-and-repurposing), uses AI to compute all-by-all treatment likelihood scores between a list of approved drugs and a list of diseases to generate new repurposing hypotheses. To support and validate ML models for drug repurposing built with KGs, it is critical to establish a carefully curated and reliable list of approved drugs and diseases ingested as KG entries. It is also essential to annotate the ground-truth entries in the sparse matrix of all possible drug–disease combinations—i.e. the list of “positive” drug–disease pairs associated with therapeutic indications and contraindications considered as “negative” treatment associations, with all remaining drug–disease pairs serving as a possible source of novel repurposing hypotheses. It is also important to consider the drug repurposing challenge in the context of met, partially met, or unmet medical need. In this regard, Fig. 1 presents our overarching view of all possible drug–disease relationships structured by categories of medical needs (including aspects related to quality of life, QoL) and availability of drugs or drug candidates (e.g. approved, under development, *de novo* discovery). Currently, the “untreated” and “treated, potential for QOL improvement” portions of the disease axis and the approved and off-market portions of the drug axis are considered of primary importance for novel repurposing hypothesis generation.

Here, we introduce the Medicines, Diseases, Indications, and Contraindications (MeDIC) database, built directly from official regulatory data sources only. We established MeDIC as a foundational database for drug repurposing with four primary goals: first, enumerate all drugs, diseases, indications, and contraindications listed in primary federal sources from the USA and other regulatory bodies as an open and freely accessible resource; second, provide additional metadata commonly required for physicians, scientists, and data scientists pursuing drug repurposing; third, make this resource user friendly, searchable, and machine-readable by delivering it in a simple, harmonized tabulated format; and fourth, take advantage of modern curation tools like LLMs and automated entity linking to make MeDIC an open-source project that is updatable regularly and automatically in order to ensure its sustainability. Specifically, MeDIC is a frequently updated collection of all drugs approved by regulatory authorities in the United States, Europe, Japan, India, and Russia, along with a list of distinctly treatable diseases and all known on-label treatment indications and contraindications originating from the United States, European, and Japanese regulatory authorities. MeDIC can be readily ingested and used in downstream machine learning applications such as model training or KG creation.

## Materials and methods

Each element of MeDIC described below is generated using a data science pipeline constructed with Kedro [29]. Kedro is an open-source data science framework for creating maintainable and reproducible code, which is currently owned and maintained by the Linux Foundation.

### MeDIC drug list

The MeDIC drug list aims to provide a comprehensive list of drugs approved in humans, metadata to inform whether they are relevant for specific repurposing cases, and a mapping to ontological identities to enable downstream applications.

The drug list was generated using the following process: government drug approvals data were first directly extracted from various sources (Table 1) [30–35]. Each list was converted into a standardized tabulated format with drug names and dates of approval and de-duplicated. Drugs not intended for human use were then identified according to source metadata and eliminated from each list. Next, an LLM (GPT-4o; see the “Discussion” section) was used to tag combination therapies consisting of two or more ingredients. Any active ingredients not present as a separate entity in the drug list (e.g. clavulanic acid) were appended as separate rows. Drugs were then resolved into normalized ontological IDs using the NCATS Name Resolver (https://github.com/TranslatorSRI/NameResolution) service augmented by an automated LLM-based QC process (Supplementary Fig. S1). We found that this LLM QC method substantially improved the accuracy of entity linking: randomly sampling 100 items each from the FDA Orange Book, FDA Purple Book, EMA, and PMDA approval lists and manually annotating correct versus incorrect identification with Name Resolution alone versus Name Resolution with LLM QC, we found that accuracy increased from 88.25% to 96.75%, respectively. Remaining deficiencies mostly concern subtle differences between factor eight drugs (https://www.ncbi.nlm.nih.gov/books/NBK583270/table/pe.app1.tab3/) and other complex biologic concepts.

**Table 1.** Data sources used in the construction of the MeDIC drug list

Country / Region	Resource	Format	Reference
United States	FDA Orange Book (small-molecule therapeutics)	TXT	[30]
United States	FDA Purple Book (biologics)	TXT	[31]
Europe	EMA European Public Assessment Report	XLSX	[32]
Japan	PMDA New Drug Approvals	PDF	[33]
India	CDSCO New Approvals	JSP	[34]
Russia	State Register of Medicines	XLSX	[35]

Approval and marketing tags were then added to each identified drug (e.g. drugs extracted from the FDA Orange Book are marked under American approval, and metadata from the raw data source is used to extract the marketing status). Alternate IDs for each therapeutic concept were then added via Translator Node Normalizer (NN) (https://github.com/TranslatorSRI/NodeNormalization). NN is a service that resolves the issue of multiple representations for the same concept by separate ontologies. When a Compact URI (CURIE) from an ontological entry is fed to NN as an argument, NN returns a new CURIE that serves as the top-level identifier for that concept (“clique leader”); any other CURIE representing that concept fed into NN should in theory normalize to the clique leader. This piece of infrastructure allows us to construct MeDIC without designing a new ontology; we can instead use clique leaders to represent each concept.

This process was repeated for each individual data source, and all extracted lists were then joined by their primary identifier to form a completed drug list with approval tags for every country. Metadata were then added to each drug entry using LLMs to produce drug groupings so that drugs not fit for repurposing could be removed from the list. Allergens (e.g. peanut, cat dander, grass pollen) are not considered to have repurposing potential and were omitted from the final drug list. Diagnostic radioisotopes (e.g. technetium-99m, fludeoxyglucose-18F, and barium sulfate) are not considered to have significant repurposing potential and were removed from the final list. Other compounds with little or no repurposing value *per se* (e.g. water, hydrogen peroxide, phospholipid) were also removed from the final list. The filters to remove drugs with little repurposing value are applied to the list, and then the respective columns are deleted. While these filters are LLM-based and therefore not guaranteed to be perfectly accurate, we have found them to substantially reduce the incidence of drug types, which are less ideal for repurposing.

Combination therapies (co-formulated combinations of multiple activated drug ingredients) are of great current and future interest but are more complex to repurpose than individual active ingredients; this tag is added to ensure physicians can identify combination therapies and filter them where it is pragmatic to do so. Following this step, ATC codes were then extracted using a strategic automated web search (Supplementary Fig. S2), and SMILES strings were extracted from PubChem where possible (for instance, no SMILES are provided for biologics or infrequent cases of drug combinations with unknown or complex chemical composition such as botanical-derived drugs). Various other tags were applied to group drugs into useful categories (Supplementary Table S1). Finally, drug lists were split according to the rigorousness of the approval agency into a “stringent approval” drug list containing entries from nations registered as WHO-listed regulatory bodies (https://cdn.who.int/media/docs/default-source/medicines/regulatory-systems/wla/list_of_wla.pdf) (currently including the USA, Japan, and Europe and in the future expanded to include resources like the British National Formulary) and “flexible approval” drug list (adding Russia and India and eventually incorporating several other regions of interest). This provides maximum flexibility according to specific repurposing projects.

**Figure 2.**
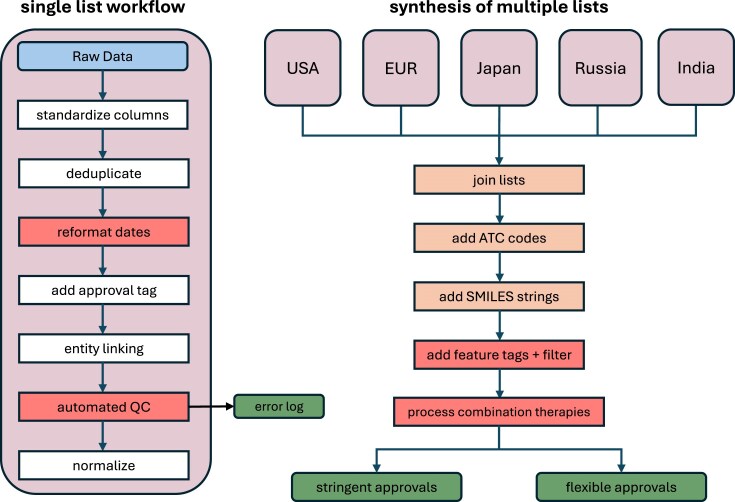
Drug list generation workflow. Steps colored in red involve the use of LLMs for automation of the list generation process. Each individual list is passed through a processing pipeline, whereby the raw data are reformatted to be compatible with the pipeline, and the list is subsequently de-duplicated, entity-linked with automated LLM-based QC, and normalized. Completed lists are synthesized into the final drug list products by joining and then adding relevant features, including ATC codes and SMILES strings to represent structure where applicable (for instance, no SMILES are obviously provided for biologics or infrequent cases of drug combinations with unknown or complex chemical composition, such as botanical-derived drugs), and various additional filters, such as drug-type classification and combination therapy component enumeration.

The output of this process is two lists of drugs (“stringent” and “flexible”) that are de-duplicated and mapped to various ontological identifiers, with all metadata displayed as columns. A flowchart of this process is shown in Fig. 2.

### MeDIC disease list

The MeDIC disease list aims to provide a comprehensive list of human diseases, enriched with metadata such as filters useful for drug repurposing, synonyms, and cross-references. The list uses the Mondo disease ontology [36] as its starting point. Mondo integrates a variety of disease ontologies and medical terminologies with a strong but not exclusive focus on genetic and rare diseases, which are of particular interest for drug repurposing. All human diseases in Mondo are included in the disease list, along with a wide variety of labels that are useful for drug repurposing-related workflows such as expert curation. Many of these labels help users with separating “true diseases” (such as “Alzheimer disease”) from grouping classes (like “neurodegenerative disorder”) or very specific disease subtypes that are not currently widely clinically diagnosed outside of highly specialized environments (e.g. “Alzheimer disease type 1”). Other columns include useful groupings (flags that state whether a disease is a cancer, an inflammatory disease, etc.), tags such as related medical specializations, and information about related disease subtypes.

Disease list metadata includes:

- a human-readable definition of the disease, synonyms, and mappings (cross-references) to other disease resources;
- labels indicating where entries represent disease groupings. These do not correspond to individual “disease entities,” but rather groups of diseases, e.g. “cardiovascular disorder” or “cancer” [37]. Heuristics that contribute to the question of whether a disease is a proper disease entity rather than a grouping include whether or not the disease has a corresponding ICD10 diagnostic code that is billable; whether the disease corresponds to a disease entity according to a disease-defining authority such as Orphanet [7] or OMIM [38]; whether a major disease curation organization (such as ClinGen [37]) has used the disease for curation of disease-causing genes and variants; or other heuristics available at docs.dev.everycure.org/pipeline/data/drug_disease_lists/;
- flags to denote whether a disease has known treatments or not;
- tags such as related medical specializations;
- expected quality-adjusted life years (QALYs) lost for sufferers: diseases with high QALYs lost may be considered higher priority for repurposing efforts; 
- information about related disease subtypes, such as counts.

**Figure 3.**
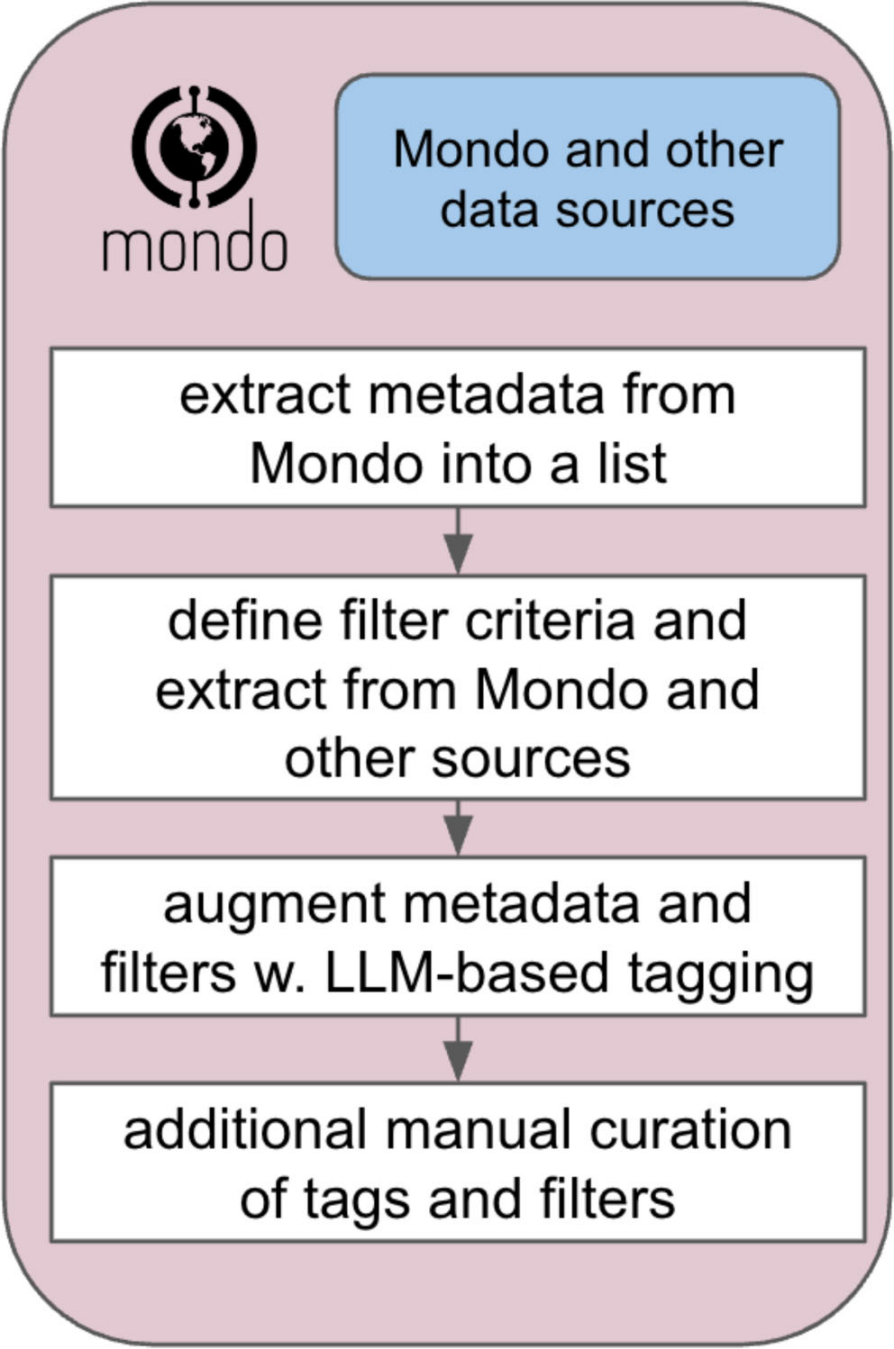
A workflow for creating MeDIC disease list. Mondo data are first extracted into a list. Filtering criteria are applied, and disease concepts not present in Mondo are extracted from other sources to arrive at a list with all relevant disease concepts.

The disease list was generated using the following process. Starting with the full Mondo disease ontology, a list of all human diseases (excluding disease susceptibilities and injuries) was created, along with disease concept level metadata such as synonyms, definitions, and cross-references that were extracted directly from Mondo. A set of predefined filter criteria (as discussed above) was then extracted from the ontology. Some of these filter criteria such as “billable ICD10 code” required the integration of external resources (such as the “Centers for Medicare & Medicaid Services”), but many of the features for filtering could be directly obtained from Mondo, which provides rich metadata on authoritative resources and links to other databases. Additional metadata not already present in Mondo were then added using an LLM pipeline (GPT-4o-mini; see the “Discussion” section). Extracted information included the QALYs lost and known treatment flags discussed above. Additional tags were then added as needed, either using LLM-based strategies or expert curation and community feedback. For example, we use a mix of LLMs and expert curation to group diseases according to an important recent holdout experiment in the drug repurposing domain (zero-shot repurposing/TxGNN [14]), so that the experiment can be replicated with our own datasets. A flowchart of this process can be found in Fig. 3. The disease list is exported as a spreadsheet with all metadata exported as columns.

The disease list is actively maintained by a team of experts and remains synchronized with the evolving Mondo ontology. Updates occur quarterly and reflect additions, removals, and reclassifications in Mondo. The list is open to community feedback (see Data availability).

### MeDIC indication and contraindication list

The goal of the indications list was to provide a comprehensive list of drug–disease pairs where each drug has been approved specifically to treat that disease. The goal of the contraindication list was to provide a comprehensive list of diseases and drugs that have been specifically flagged as medically contraindicated (e.g. likely to cause or worsen a disease itself or otherwise not recommended for use in patients with the disease).

For repurposing use cases, drugs commonly used “off-label” (e.g. drug indications that are not formally approved but are commonly prescribed) are also of interest. While this was out of scope for this work, it is being explored by Every Cure going forward.

Indications were extracted from the sources listed in Table 2 [32, 33, 39]. Each list was processed using the same data science pipeline. The raw data was first converted into a structured data frame with rows for active ingredients, natural language indications text, and provenance linking to the data source. We intentionally maintained abstraction of drug active ingredients to the active moiety level rather than maintaining links to specific formulations and dosage (if present in the indication), as such types of details are provided by physicians in prescriptions on a patient-by-patient basis. As of v1.0 of MeDIC, contraindications are only extracted from FDA labels. Each entry’s natural language indications or contraindications text was processed using an LLM (Gemini 2.0; see the “Discussion” section) to generate a structured list of diseases treated.

**Table 2.** Indication list data sources

Country	Source name	Format	Reference	Comments
United States	DailyMed drug labels	XML	[39]	Structured Product Labels (>50 000 files)
Europe	European Public Assessment Report	XLSX	[32]	“Therapeutic Indication” section
Japan	New Drug Approvals	PDF	[33]	PDF first converted to CSV, therapeutic indications section for each drug entry

Contraindications were sourced solely from US drug labels.

This process yielded a data frame with four columns: source file, active ingredients, indications text, and a structured list of diseases treated by the drug according to the drug label. This data frame was then “flattened” to ensure only one drug–disease concept pair was contained per row; indication text was retained for provenance. NCATS/RENCI Name Resolver [40] was used to resolve drug and disease names into ontological IDs on this flattened list. We note that, as discussed by Nelson *et al.* [41] and Moodley *et al.* [42], there does not exist an ontology with sufficient precision to establish the full medical context of every therapeutic indication. Details such as prior interventions, disease severity or stage, genetic mutations, observed analytes and clinical laboratory test results, and patient type (adult, pediatric, pregnant, nursing, etc.) are often excluded from these ontologies. Indeed, it is impractical to map every permutation of every disease owing to the combinatorial nature of these details. We thus rely on current ontologies and allow physicians to consult guidelines such as UpToDate [43] or the National Comprehensive Cancer Network guidelines [44] for complete medical context. We partially rectify this with the addition of hyperrelations, or “connections-to-connections” linking drug-disease connections to additional ideas or concepts that fully define the relationship. These were also added via LLM (see the “Discussion” section). For example, for the case of Rituximab, additional hyperrelation information such as (Rituximab – treats → Wegener’s Granulomatosis) – when used in conjunction with → glucocorticoids and (Rituximab – treats → Wegener’s Granulomatosis) – in subpopulation → adult patients was added automatically. Hyperrelations can be used to describe most additional context qualifying the drug-disease connection. The overarching context for each drug label is present in an easily ingestible JSON format, presented online with each drug–disease link for additional context. While these additional details cannot yet be used for machine learning-based drug repurposing predictions to distinguish between the efficacy of a drug on, e.g., cancers with a specific mutation vs. an unmutated instance, there exist methods that may be used to exploit these hyperrelations [45].

As in the drug list, an automated LLM-based (OpenAI-GPT-4o) QC workflow was then used to verify correct resolution of the ontological ID and choose a better one when correct identification could not be achieved on the first attempt (Supplementary Fig. S1). The matching ID was then passed through NCATS Node Normalization service to guarantee that the concept could be correctly linked to any knowledge source or KG that has been normalized using the same service. This step also enables resources that have been normalized using older versions of Node Normalizer to still make use of this list by renormalizing both resources. Where an indication or contraindication was identified for diseases containing subclasses, a tree down-filling algorithm was used to descend the MONDO hierarchy and infer edges between the drug and all of the top-level disease’s descendants for each indication in the base indication list. Critically, these inferred edges must not be interpreted in any way as on-label or approved indications or contraindications; these are provided solely for research or informational purposes and have been clearly marked as such (including the file names in capital letters) in the MeDIC release documents online. This expanded list is registered separately from core on-label indications to ensure provenance can be tracked. We suggest that this down-filling may be of benefit to data scientists where additional training data can improve predictive capability of machine learning models.

**Figure 4.**
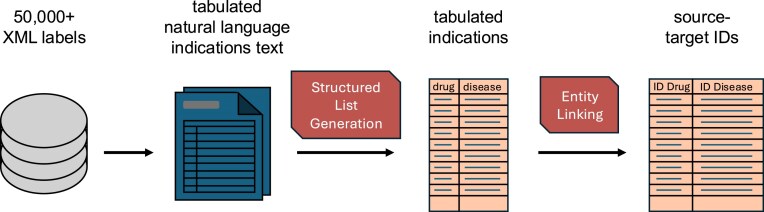
MeDIC on-label indication and contraindication workflow. All FDA drug labels are extracted from the DailyMed database [39]. Indication text is extracted, and all >50 000 files are converted to tabulated format. These tabulated indications are identified using entity linking and LLM-based automated QC, returning a list with source and target IDs associated with therapeutic indications. For European and Japanese lists, processing begins at the “tabulated natural language” stage using intermediate products from the drug list to retain mapping parity between listed drugs and therapeutic indications. Lists are finally joined to produce the final indication list. Contraindications are currently sourced solely from FDA drug labels.

The output of this process is four lists: a tabulated indication list where each row contains a drug, disease, their respective labels, and hyperrelations, linking back to source text; a contraindication list containing the same; and down-filled indications and contraindication lists where their parent list has been down-filled to add inferred drug–disease connections. Fig. 4 describes the construction of the indications and contraindication lists.

### Physician review

Physician review was iteratively used to ensure the automated generation workflow outputs, such as LLM-enabled text processing and ontological ID mapping, were reflective of the upstream sources and helpful to discover new disease treatments. Identification of issues through manual review helped refine the LLM prompts and clarify both inclusion and exclusion criteria. Such issues included the presence of non-therapeutic drugs and multiple salts of the same drug in the drug list, as well as incorrectly labelled indications in the ground truth lists. Validation of a random sample of 100 random indications text sections from US FDA drug labels revealed 96% of indications sections were extracted in a fully satisfactory manner, with the four failing items either including a disease that was mentioned in the text but not indicated or generating additional indications for the drug that were not explicitly present in the text (see Supplementary data).

## Results

### Drug list

**Figure 5.**
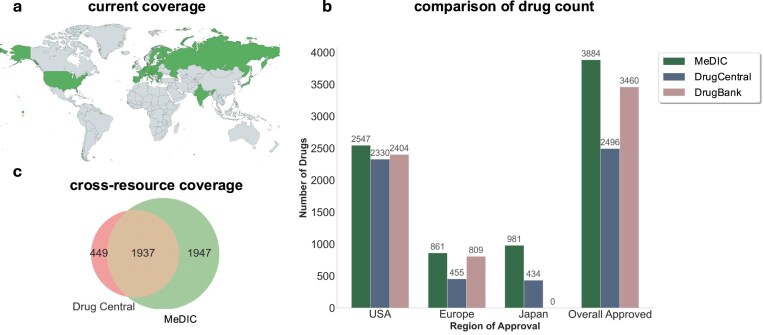
Drug list coverage map and comparison between MeDIC and existing sources. (**A**) A map highlighting geographic locations of current nations or regions covered by the MeDIC drug list. (**B**) Drug counts for the USA, Europe, Japan, and the overall count of approved drugs in MeDIC after filtering out allergens and vaccines in comparison with similar contents in Drug Central and DrugBank. Drug Central values were extracted from the downloadable lists for US, European, and Japanese approved drugs on their core website. DrugBank values were extracted using the filters on their website. (**C**) Mapping coverage between Drug Central and MeDIC. Approval tables for the US, Europe, and Japan in Drug Central were joined and normalized, and the sets of drugs from MeDIC and Drug Central were compared.

The first outcome of MeDIC was the generation of a list of approved pharmaceutical products linked directly to their respective regulatory approval sources. Two lists are provided: the “stringent” list contains only drugs approved by more restrictive approval agencies according to the WHO, currently including the US, Europe, and Japan, and an expanded list contains additional approved drugs from India and Russia. The latter list can be expanded to include other regions of interest (Fig. 5A).

The stringent list contains 2836 drugs, and the expanded list contains 3883 drugs. The expanded approvals list contains 664 combination therapies and 673 biologics, while the stringent approval list includes 444 combination therapies and 548 biologics. Both lists contain harmonized identities for each concept so that the drugs can be ingested easily within a normalized KG. Multi-component therapies contain IDs for their individual components and also allow for easy construction of edges between combination therapies and their ingredients. This may aid in future novel combination therapy nomination projects. Both lists contain 21 filtering options (Supplementary Table S1), which can be used to select for different approval regions, marketing statuses, drug types, and formulation styles (e.g. selecting only single therapies). An additional ATC code [46] feature provides these codes and each level of ATC hierarchy for easy filtering on over 2100 drugs. SMILES strings [47] are also provided where available, covering 2636 drug concepts representing most small molecule therapies (most combination therapies and biologics are not considered). A breakdown of individual country statistics compared to other common drug resources can be found in Fig. 5B and C quantifies cross-resource coverage with Drug Central.

The main driver in the difference between total drug counts in MeDIC as compared to existing databases is the inclusion of combination therapies. DrugBank and Drug Central describe links between the names of combination therapies and their components, but combination therapies themselves are not top-level entries. Based on MeDIC counts, combination therapies constitute over 15% of all approved medications and ~20% of medications approved in the USA. While many of these reflect highly similar OTC combinations or combinations designed for patient ease (for example, amlodipine and valsartan: two antihypertensives commonly taken concurrently), there are cases where combination therapies offer genuine mechanistic synergy (for example, beta-lactamase inhibitors prescribed in tandem with antibiotics serve to combat drug resistance [48]). In addition, linking combination therapies to their components can provide valuable additional context when using this information for machine learning tasks. We have therefore included combination therapies in the MeDIC drug list.

### Disease list

The second outcome of MeDIC was the generation of the MeDIC disease list comprising 22 779 disease concepts. Given that the concept of “disease entity” depends on the exact circumstances (some clinicians may perceive “breast cancer” as a clinically actionable disease entity, while others would want to distinguish different kinds of breast cancers like carcinoma and sarcoma), we developed a set of clearly defined metadata specifically curated to provide categories of disease that are helpful for assessing drug repurposing candidates and can therefore be used to create a bespoke disease list (for example, to prioritize the review of viable drug-repurposing candidates by clinicians).

### Indication and contraindication list

**Figure 6.**
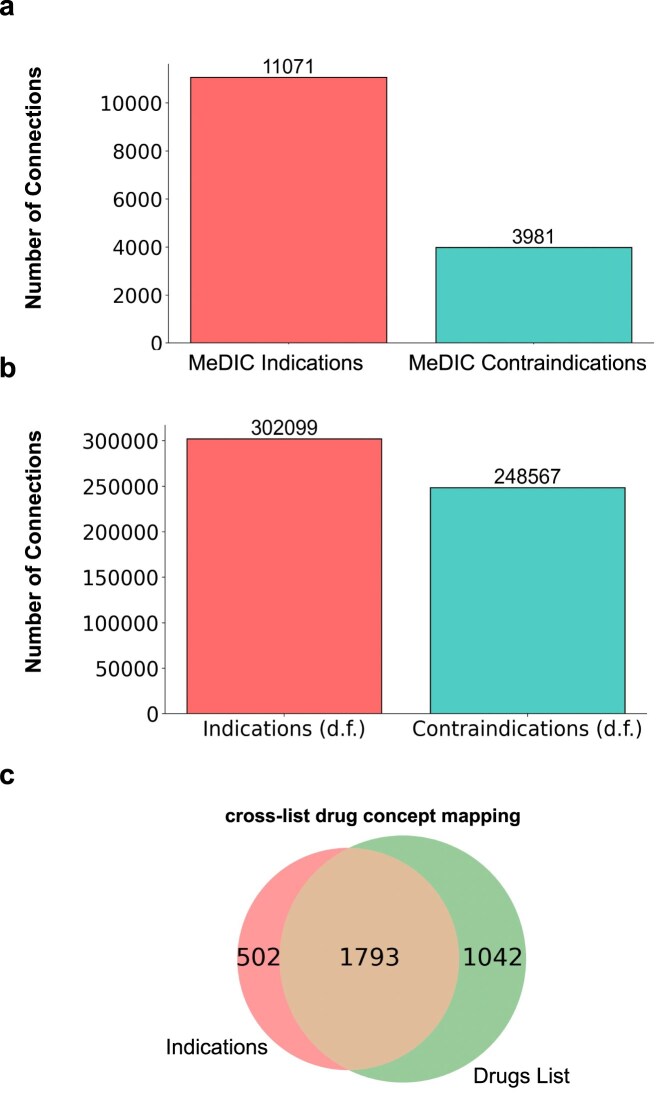
Indication and contraindication list statistics. (**A**) Un-down-filled indications and contraindications total quantities. (**B**) Down-filled (d.f.) indications and contraindication list total quantities. (**C**) Cross-product mapping between the MeDIC drugs list and the MeDIC indication list.

The third outcome of MeDIC was the creation of a list of connections between drug and disease concepts in the context of treating or being contraindicated for such diseases, including which regulatory authority has determined each relationship. These indications and contraindications are extracted directly from approval resources with provenance back to the documents that they were extracted from, making it easy to fact-check each entry. We assessed 100 randomly sampled indications from the extraction list manually and found 96% of them to correctly address all indications in the text (see Supplementary File S1), with the condition of correctness being that (i) the indication is not incorrect based on expert opinion and (ii) a physician can use relevant medical guidelines to understand the full medical context of the drug-disease pair’s relationship. The core indication list contains 11 068 indications spread over 2295 unique drugs and the core contraindication list contains 3982 contraindications spread over 994 unique drugs (Fig. 6A). The expanded down-filled indication list contains 302 000 entries, and the down-filled contraindication list contains 248 000 entries (Fig. 6B). As stated above, the expanded lists are intended for research purposes only. To the best of our knowledge, we report the first use of hierarchical disease ontologies to connect drug and disease entities. We find that the drugs list overlaps substantially with the indication list, but there are still some entities missing (Fig. 6C) (see the “Discussion” section).

## Discussion

MeDIC provides comprehensive and up-to-date information about approved drugs and their established therapeutic uses to medical, patient, and research communities. MeDIC enables multiple uses such as data exploration stratified by disease, drug type, and approval region. Exploration of MeDIC allows investigators to highlight diseases that have adequate therapeutic options as well as those still presenting an unmet medical need, including diseases with no current approved pharmacotherapy. Finally, MeDIC provides a fit-for-purpose, highly curated knowledge base to train and evaluate ML and AI models for nominating drug repurposing candidates.

One limitation of the overarching approach to omitting an ontology and performing entity linking to existing ontological entries is that entity linking can occasionally lead to the resolution of concepts into a “lonely” ID that represents the correct concept but has poor connectivity to other parts of a correctly normalized KG (e.g., as of August 2025, UNII:I47IU4FOCO (WARFARIN POTASSIUM) exists as a distinct clique from CHEBI:10033 (Warfarin) despite drug-chemical conflation being used in normalization, where all formulations and salts of an active ingredient are supposed to be joined into a single clique). As we find these issues, we feed them upstream into the tools used for name resolving and normalization, which ultimately positively impacts list quality. Additionally, while MeDIC contains an overall larger number of approved drugs than existing databases, it is focused on approved drugs that are relevant to a repurposing use case. It therefore currently does not include items such as the FDA “Generally Regarded as Safe" list, herbal medicines registry, and the pediatric investigations registry. The listed approved drugs in MeDIC should be regarded as more suitable for repurposing, rather than more comprehensive, than existing databases.

A substantial portion of the discrepancy between Drug Central and MeDIC drug lists (Drug Central drugs missing from our list, Fig. 5C) is explained by the omission of radioisotopes and allergens; we did not have the ability to filter these concepts in Drug Central, and so they were left in. Other discrepancies exist where identical concepts have different mappings in Drug Central versus MeDIC. Planned near-term improvements to NN may resolve some of these discrepancies as cliques become more comprehensive and drug-chemical conflation improves.

While the filtering methodology employed in the disease list provides a strong baseline, known limitations include incomplete mappings (e.g. ICD-10 coverage in Mondo), which may lead to false positives or negatives. Ongoing improvements to both heuristic design and community feedback are intended to refine the list’s utility over time.

While LLM-based indication and contraindication extraction is generally very accurate, in cases where indication classification is very subtle (e.g. vemurafenib for melanoma, where the V600E mutation is a vital factor in the correct use of the drug [49]), LLMs are sometimes unable to capture this distinction. There are also cases where indication text is confusingly or ambiguously worded and includes references to contraindications, which can, very rarely, end up on the indication list. Another occurrence is that contraindications text references patients with a certain disease for which the drug is usually indicated but contextualized by certain qualifying factors (e.g. drug *x* is contraindicated in patients with disease *y* who also have phenotypic feature *z* even though *x* is usually indicated for *y*). This results in a contraindication being registered between *x* and *y*, resulting in *x* being both indicated and contraindicated for *y*.

We find that LLMs are an extremely capable technology but should not be leaned on for ambiguous tasks. We have thus used LLMs at selective points in the list generation process where tasks were simple but laborious, therefore saving curators significant time. For example, some regulatory authorities have inconsistent date formatting on their approval lists (e.g. India). Using LLMs at low temperature for this task has an extremely high accuracy and safely ignores things like extra text in the field. Similarly, many lists were found to have inconsistent delimiting between combination therapy ingredients and sometimes introduce a delimiter in the middle of an ingredient name, leading to defective ingredient splitting; using LLMs to reformat these therapies with a consistent delimiter reduces pipeline complexity and provides robustness for processing unseen data as new regulatory data is released and MeDIC is updated. We believe that partitioning work into “LLM-Comprehensible” and “LLM-Incomprehensible” tasks and assigning LLMs where algorithmic solutions are inelegant or ineffective strikes an ideal balance between minimizing manual labor and maximizing accuracy and product quality. Simple “yes/no” questions based on indisputable fact (other than counting tasks), basic processing of lists with ambiguous formatting, and selection of candidates best meeting a set of criteria from a list of options are all tasks that LLMs handle gracefully and can be composed into complex data processing pipelines.

One example in which thoughtful implementation of LLM workflows significantly increased the quality of our output was LLM-based improvement of entity linking (Supplementary Table S2). Prompt engineering was used throughout the development of these methods to minimize erroneous or ambiguous outputs. In addition, ongoing use and curation by repurposing- oriented users (such as the Every Cure team) provides a source of feedback that is used to correct and improve each output going forward. Finally, per-item output is also kept low, and so compute usage is minimized by this approach.

OpenAI’s GPT-4o and Gemini 2.0 were both used at different stages of this process, according to cost and ease of use. Simple experimentation during method development suggests that any reasonably capable LLM could be leveraged for similar results. We found some models (such as GPT-4o-mini) were unable to consistently handle tasks that required more advanced reasoning (e.g. Boolean tagging of glucose-regulating drugs). An organization looking to optimize costs could likely distinguish between simpler and more complex tasks and select models accordingly, but the relatively low scale of total API calls required to replicate this methodology means this is unlikely to be a meaningful optimization.

It is important to highlight the differences in the contents provided by MeDIC in comparison with well-known sources such as DrugBank and Drug Central (Fig. 5) that relate largely to the approach we took to construct MeDIC. The entities in the drug, indication, and contraindication lists were harmonized using node normalization. This serves as an excellent method for joining multiple resources together and for harmonizing the lists with KGs and other sources by normalizing both the lists and the graph. This also means we do not need to maintain an ontology to keep MeDIC in service, which substantially reduces the burden of list maintenance. However, this comes with the tradeoff of not having the same long-term guarantee of ID stability as in other resources which have produced their own mini ontologies to stabilize the IDs that refer to certain drugs. This results from a combination of entity linking changes and normalization changes as well as natural randomness inherent to the use of LLMs for list processing, although the latter has been largely mitigated through the use of low model temperatures and precise prompting. This also explains, for example, the imperfect mapping between Drug Central and the MeDIC drug list. One method to resolve this in the future could be to establish a record of how the primary ID of the drug concept has changed over time so that a user ingesting a prior version of the drug list can easily produce a mapping to the current version of the drug list for downstream applications. Another method could be to simply normalize all resources including records prior to modelling, as this would guarantee dynamic attachment of previous results to current results as long as entities have not been deprecated.

Future updates to MeDIC will include additions of disease phenotypes, known biological targets of drug action, and off-label uses extracted from clinical data to further support a multitude of research efforts in drug discovery and repurposing, as well as patient subgroup stratification for pediatric uses.

### Use of MeDIC for ML-based drug repurposing

**Figure 7.**
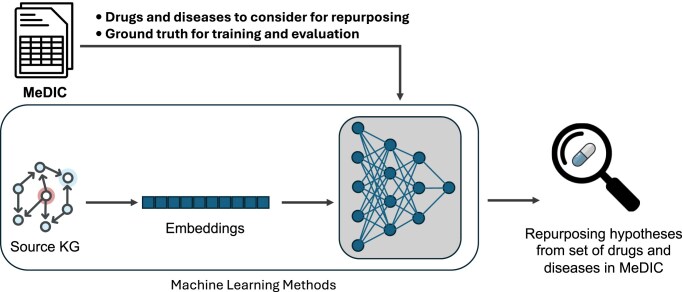
Implementation of MeDIC as a drop-in data source to solve the problem of which drugs can be repurposed, which diseases to evaluate them against, and which data can be used for validation upon model development. This is shown in the context of a standard embedding and downstream ML application, where node embeddings from the KG representing drugs in the MeDIC drug list and embeddings representing the diseases in the MeDIC disease list are fed through a deep neural network for repurposing hypothesis generation.

As mentioned in the introduction, any drug repurposing hypothesis generation requires a list of approved drugs (as the objects that can be repurposed). In addition, known relationships between drugs and diseases are required for training and/or evaluation purposes. Finally, a list of target diseases is required. MeDIC fulfills these requirements while retaining an open format. Further, many AI-drug repurposing projects use knowledge graphs (KGs) as input. Most large-scale biomedical KGs are relatively indiscriminate in their classification of “drugs,” so, while it is possible to assemble a list of drugs directly from the KG, this is usually too broad and results in nominations of mostly non-approved compounds (or, in the case of unconflated KGs, dozens of formulations representing the same active moiety concept). Similarly, treatments, or “treats” connections between drugs and diseases registered in the KG, are often too promiscuous to be used as validation data. Any existing KG can be normalized with the same tools used to build MeDIC, and the two can be used together seamlessly, or MeDIC can be adapted to whatever schema is used by the KG. One potential application of MeDIC as a curated ground-truth source for drug repurposing hypothesis generation using KGs is depicted in Fig. 7.

## Supplementary Material

gkaf1312_Supplemental_Files

## Data Availability

MeDIC is publicly available at its repositories and at medic.renci.org. All code for list generation is hosted at https://github.com/everycure-org/medic. The disease list is currently archived at https://doi.org/10.6084/m9.figshare.30491108. The indication list and contraindication list are currently archived at https://doi.org/10.6084/m9.figshare.30491081. The drug list is currently archived at https://doi.org/10.6084/m9.figshare.30491141. All lists are open to community feedback. Comments on each list can be registered in GitHub.
