# Supplementary material for: Medicines, Diseases, Indications, and Contraindications (MeDIC): a foundational resource to support drug repurposing
Source: Nucleic Acids Res. 2025 Dec 12;54(D1):D1477–87. doi: 10.1093/nar/gkaf1312 (PMC12807713; doi:10.1093/nar/gkaf1312)
Supplement: gkaf1312_Supplemental_Files [file gkaf1312_supplemental_files.zip › MeDIC Supplement.pdf]

Medicines, Diseases, Indications, and Contraindications (MeDIC): A Foundational Resource to Support Drug Repurposing

SUPPLEMENTARY DATA

Marcello DeLuca[1], Nico Matentzoglu[2], Elliott Sharp[3], Jane Li[3], Piotr Kaniewski[3], Kathleen Carter[4], Kushal Koirala[1], Elvin Ding[1], Laurens Vijnck[3], Pascal Brokmeier[3], Sabrina Toro[5], Kevin Schaper[5], May Lim[3], Charlie Hempstead[3], Jacques Vergine[4], Olivia Li[1], David C. Fajgenbaum[3,6], Christopher Bizon[4], Melissa Haendel[5], and Alexander Tropsha[1,4]*

[1] Division of Chemical Biology and Medicinal Chemistry, University of North Carolina Eshelman School of Pharmacy, Chapel Hill, North Carolina 27599, USA

[2] Semanticly, Athens 10563, Greece

[3] Every Cure, Philadelphia, Pennsylvania 19104, USA

[4] Renaissance Computing Institute, Chapel Hill, North Carolina 27517, USA

[5] Department of Genetics, University of North Carolina School of Medicine, Chapel Hill, North Carolina 27599, USA

[6] Perelman School of Medicine at the University of Pennsylvania, Philadelphia, Pennsylvania 19104, USA

*To whom correspondence should be addressed. Tel: **+1 919 966-2955**; Email: **alex_tropsha@unc.edu**

Supplementary Table 1: Filtering Options for MeDI Drug List

Filter Name	Type	Description
approved_usa, approved_europe, approved_japan, approved_india, approved_russia	bool	Drugs approved in the region of interest
marketing_status_usa	bool	For drugs approved in the USA, the most permissive formulation of that active moiety (OTC, RX, or Discontinued)
is_combination_therapy	bool	Whether the drug is or is not a coformulation of multiple active moieties
combination_therapy_ingredients	list	List of active ingredients, if a combination therapy
combination_therapy_ingredients_curies	list	List of primary IDs associated with all active moieties in the combination therapy
is_steroid	bool	Describes whether or not the drug is a steroid
Is_antimicrobial	bool	Describes whether or not the drug is classed as an antibiotic, antiviral, antiretroviral, antiparasitic, or antifungal.
is_chemotherapeutic	bool	Describes whether or not the drug is used as a primary chemotherapeutic for cancer therapy
is_glucose_regulator	bool	Describes whether or not the drug functions as a glucose regulator
is_vaccine_or_antigen	bool	Describes whether or not the drug functions as a vaccine or antigen
is_metallic_salt	bool	Describes whether the active moiety is a simple metallic salt
is_allergen	bool	Describes whether the drug is an allergen used in allergy testing
is_radioisotope_or_diagnostic_agent	bool	Describes whether the drug is a radioisotope used primarily for diagnostic purposes
is_cancer_drug	bool	A more broad classification than chemotherapeutics describing any drug used for the treatment of or in conjunction with another drug for the treatment of cancer, e.g., to relieve cancer treatment related symptoms.
alternate_ids	list	A list of all alternate IDs from ontologies other than that of the primary identifier which describe the same concept
atc_codes	list	Anatomical Therapeutic Classification code
smiles	string	SMILES string

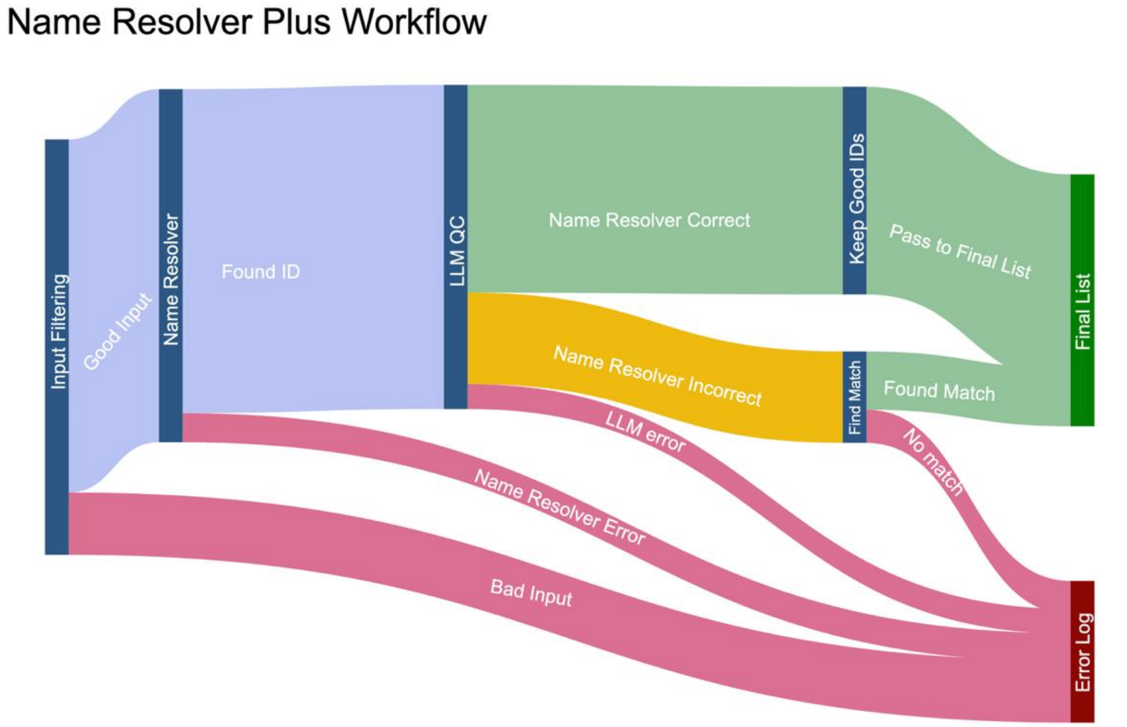

Supplementary Figure 1: Automated QC workflow using LLMs to verify and improve the accuracy of the entity resolving processes. Inputs are first filtered for mis-formatted data, errors from previous steps, or blank input. Name Resolver is then used for a first attempt at entity linking. LLM-based comparison is then used to compare the input concept to the label of the linked entity. If these concepts match, the linked ID is kept and passed to the final list. If they do not match, the concept is re-resolved and an LLM is asked to either choose a matching concept from the top 30 results or to indicate that no matches were found in the top 30 results. If a match is found, the linked entity is passed onto the final list. If no match is found, the concept is passed into the error log. All failures are recorded to provide a metric of list generation pipeline health.

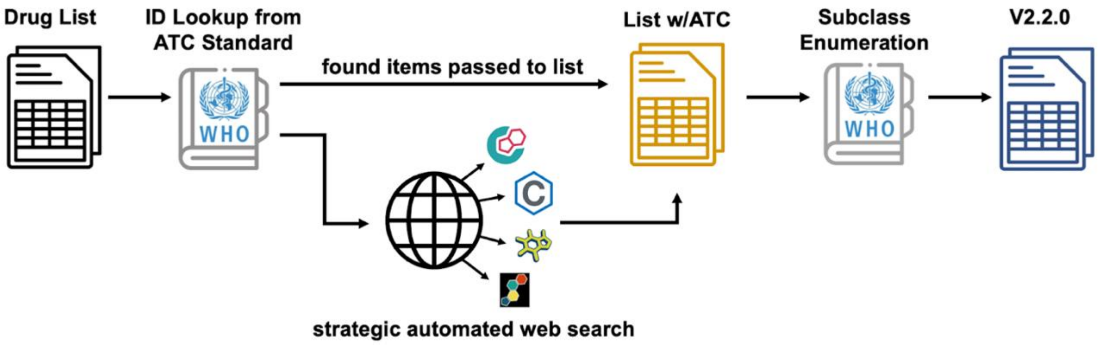

Supplementary Figure 2: ATC Code Lookup Feature for MeDI List. The drug list is fed through a dictionary of reference IDs in the ATC standard documents; if found, these items are passed directly into the ATC-augmented drug list. If not found, the drug ID is fed into ChEMBL, CHEBI, PubChem, and Drug Central in search of cross-referenced ATC code values. Following the search for ATC codes, ATC subclass enumeration is performed using the ATC document as a reference. Finally, the full, subclass-enumerated list is returned.

Supplementary Table 2: Examples of LLM-based QC improving entity linking quality

Disease Text	Original Name Resolution Label	Improved Label
hypertension	portal hypertension	hypertension
clostridium	Clostridium difficile colitis	Clostridium infectious disease
hepatic disease	cyanosis and hepatic disease	liver disorder
\ depression	postpartum depression	Depressed mood
manicdepressive disorders	voice disorders	mood disorder
wolffparkinsonwhite syndrome	Sjogren syndrome	Wolff-Parkinson-White syndrome
acute nonspecific tenosynovitis	acute myeloid leukemia	tenosynovitis
stevensjohnson syndrome	Sjogren syndrome	Stevens-Johnson syndrome
infections of the skin and skin structure	skin carcinoma in situ	skin infection
